# Predictive scoring systems for in-hospital mortality due to acutely decompensated liver cirrhosis in Indonesia

**DOI:** 10.1186/s12876-021-01972-6

**Published:** 2021-10-20

**Authors:** Saut Horas H. Nababan, Arif Mansjoer, Achmad Fauzi, Rino A. Gani

**Affiliations:** 1 Hepatobiliary Division, Internal Medicine Department, Cipto Mangunkusumo National General Hospital, Faculty of Medicine, Universitas Indonesia, Jl. Diponegoro No. 71, Jakarta, 10430 Indonesia; 2 Clinical Epidemiology Unit, Internal Medicine Department, Cipto Mangunkusumo National General Hospital, Faculty of Medicine, Universitas Indonesia, Jl. Diponegoro No.71, Jakarta, 10430 Indonesia; 3 Gastroenterology Division, Internal Medicine Department, Cipto Mangunkusumo National General Hospital, Faculty of Medicine, Universitas Indonesia, Jl. Diponegoro No.71, Jakarta, 10430 Indonesia

**Keywords:** Liver cirrhosis, Acute decompensation, In-hospital mortality, Scoring system, Prognosis

## Abstract

**Background:**

Acutely decompensated liver cirrhosis is associated with high medical costs and negatively affects productivity and quality of life. Data on factors associated with in-hospital mortality due to acutely decompensated liver cirrhosis in Indonesia are scarce. This study aims to identify predictors of in-hospital mortality and develop predictive scoring systems for clinical application in acutely decompensated liver cirrhosis patients.

**Methods:**

This was a retrospective cohort study using a hospital database of acutely decompensated liver cirrhosis data at Cipto Mangunkusumo National General Hospital, Jakarta (2016–2019). Bivariate and multivariate logistic regression analyses were performed to identify the predictors of in-hospital mortality. Two scoring systems were developed based on the identified predictors.

**Results:**

A total of 241 patients were analysed; patients were predominantly male (74.3%), had hepatitis B (38.6%), and had Child–Pugh class B or C cirrhosis (40% and 38%, respectively). Gastrointestinal bleeding was observed in 171 patients (70.9%), and 29 patients (12.03%) died during hospitalization. The independent predictors of in-hospital mortality were age (adjusted OR: 1.09 [1.03–1.14]; p = 0.001), bacterial infection (adjusted OR: 6.25 [2.31–16.92]; p < 0.001), total bilirubin level (adjusted OR: 3.01 [1.85–4.89]; p < 0.001) and creatinine level (adjusted OR: 2.70 [1.20–6.05]; p = 0.016). The logistic and additive scoring systems, which were developed based on the identified predictors, had AUROC values of 0.899 and 0.868, respectively.

**Conclusion:**

The in-hospital mortality rate of acutely decompensated liver cirrhosis in Indonesia is high. We have developed two predictive scoring systems for in-hospital mortality in acutely decompensated liver cirrhosis patients.

**Supplementary Information:**

The online version contains supplementary material available at 10.1186/s12876-021-01972-6.

## Background

Globally, from 2007 to 2017, there was a 15% increase in mortality due to liver cirrhosis and chronic liver disease[1]. During the 2000–2015 period, there was an increase in the incidence of liver cirrhosis in the Asia Pacific region, including Indonesia [2]. In contrast with that in Japan, South Korea, and China, which experienced a decrease in mortality due to liver cirrhosis, mortality is increasing in Indonesia [3]. In addition, cirrhotic patients have a lower productivity level and a lower quality of life than people without cirrhosis [4].

Liver cirrhosis is the final stage in the natural course of chronic liver disease. Prognostic studies show that cirrhotic patients are heterogeneous, with different survival rates. A total of 5%–7% of patients with liver cirrhosis at the compensated stage will experience acute decompensation within one year [5]. The in-hospital mortality in acutely decompensated liver cirrhosis patients has been reported to vary from 4% to 11.6% due to differences in study designs, etiology, and drug availability [6, 7]. Several factors were associated with in-hospital mortality among acutely decompensated liver cirrhosis patients, such as demographic factors, degree of liver dysfunction, complications associated with portal hypertension, and extrahepatic organ dysfunction. However, the effect of these factors independently on mortality is not yet fully understood. Other predictors of mortality have also been studied, such as the presence of comorbidities, bacterial infection, markers of infection, such as C-reactive protein and procalcitonin, and hyponatremia [8–12].

Several scoring systems have been developed to predict mortality in patients with acutely decompensated liver cirrhosis, such as the Child–Pugh score and Model for End-stage Liver Disease (MELD). These scoring systems are effective in predicting 3- and 6-month mortality but less accurate in predicting in-hospital mortality [13]. The MELD-Na score can predict in-hospital mortality in liver cirrhosis patients in an intensive care setting with modest accuracy (area under the receiver operating characteristics curve (AUROC): 0.77–0.81) [14]. The chronic liver failure-acute decompensation (CLIF-AD) scoring system has been developed to predict short-term and long-term mortality in acutely decompensated liver cirrhosis patients, but it has not been validated in our population [7].

Liver cirrhosis is still an important health issue in Indonesia. According to the Indonesian national health insurance program, liver cirrhosis is one of the diseases with a catastrophic cost expenditure [15]. However, data on in-hospital mortality of acutely decompensated liver cirrhosis in Indonesia are still limited. This study aims to identify predictors of in-hospital mortality and develop predictive scoring systems for clinical application in acutely decompensated liver cirrhosis patients.

## Methods

This study was performed in accordance with the ethical principles of the Declaration of Helsinki (7th revision, 2013). This study was approved by the Ethics Committee of the Faculty of Medicine, Universitas Indonesia (No.KET-1368/UN2.F1/ETIK/PPM.00.02/2019) and the Institutional Review Board of Cipto Mangunkusumo National General Hospital (No.LB.02/221/0107/2020). This was a retrospective cohort study of liver cirrhosis patients aged ≥ 18 years with acute decompensation at Cipto Mangunkusumo National General Hospital, a tertiary referral hospital in Jakarta, Indonesia, between January 2016 and December 2019. All patient’s data were extracted from the hospital paper-based and electronic health records. All clinical variables, including also laboratory test, imaging, biopsy and endoscopy results were identified. The diagnosis of liver cirrhosis was based on liver biopsy or by a combination of clinical examination, imaging (ultrasound, CT/MRI, transient elastography), and laboratory results. Acute decompensation was defined as (1) grade 2–3 ascites that occurred for the first time or recurred after improvement with previous therapy; (2) hepatic encephalopathy that occurred for the first time or recurred after improvement with previous therapy; and (3) gastrointestinal bleeding secondary to portal hypertension, including esophageal and gastric variceal bleeding. Of the 894 patient records, 302 showed incomplete or lost data. So, we reviewed 592 acutely decompensated liver cirrhotic patients. The exclusion criteria were pregnancy, HIV coinfection (n = 31), immunosuppressive treatment (n = 5), advanced hepatocellular carcinoma (beyond the Milan criteria) (n = 255), postoperative or post-liver transplant, hospitalization for only diagnostic purposes or elective procedures, and hospitalization for less than 24 h (n = 60). A total of 241 patients were included in the final analysis (Additional file 1: Figure S1). Thirteen clinical variables were recorded at hospital admission: (1) age, (2) number of Charlson comorbidities, (3) history of decompensation, (4) hepatic encephalopathy, (5) bacterial infection, (6) mean arterial pressure (MAP), (7) SpO_2_/FiO_2_ ratio, (8) neutrophil count, (9) sodium level, (10) albumin, (11) total bilirubin, (12) creatinine, and (13) prothrombin time. Bacterial infections were defined according to the conventional criteria as previously reported [16], after a detailed review of all diagnostic components of the health records.

All patients with acutely decompensated liver cirrhosis received standard medical therapy based on international consensus. Patients with ascites underwent ascitic tap and ascitic fluid analysis. Patients with gastrointestinal bleeding received vasoactive therapy, blood transfusion, and endoscopy. Ligation was performed for oesophageal varices, and cyanoacrylate glue injection was performed for gastric varices. Patients with hepatic encephalopathy received lactulose therapy. Empirical antibiotic administration was based on the local guidelines for the use of antibiotics issued by Cipto Mangunkusumo National General Hospital. None of the patients underwent the transjugular intrahepatic portosystemic shunt (TIPSS) procedure. None of the patients received liver-support therapy, such as plasmapheresis, or underwent liver transplantation.

### Statistical analysis

All data were validated before they were processed using STATA software (release 15.0, STATA Corporation, College Station, TX). Continuous variables are presented as medians (minimum–maximum) or means ± standard deviations, as appropriate. Categorical variables are presented as frequencies (percentages). Bivariate simple logistic regression and multivariate logistic regression analyses were performed to identify independent predictors of in-hospital mortality. For the development of predictive scoring systems, variables with a *p*-value of < 0.25 in the bivariate analysis were entered into the multivariate logistic regression analysis using the backward stepwise method. The performance of the scoring systems was evaluated by the Hosmer–Lemeshow test and visually presented as a calibration plot. The discriminatory power was evaluated by the area under the ROC (AUROC) curve. Using receiver operating characteristic (ROC) curve analysis, the cut-off point with optimum sensitivity and specificity to predict in-hospital mortality was determined. The discrimination ability of our scoring systems was further assessed by comparing its AUROC with those of other scores such as MELD, MELD-Na and CLIF-C OF. International normalized ratio (INR) was not available for 56 patients. Since INR values correlated well with prothrombin time ratio, INR values were imputed using regression imputation with Expectation Maximization (EM) method with prothrombin time ratio as predictor. A *p-*value of < 0.05 was considered significant.

## Results

### Baseline characteristics

The patients' baseline characteristics and comparisons between survivors and nonsurvivors are presented in Table 1. In total, 241 patients were included, and 29 patients (12.03%) died during hospitalization. A total of 74.3% of patients were male, with a mean age of 53 years. The most common etiologies were hepatitis B (38.6%) and hepatitis C viruses (29%). Most of the patients were classified as having Child–Pugh class B or C cirrhosis (40% and 38%, respectively), with a median score of 9. A previous history of acute decompensation was noted in 20% of the cases. The most common comorbidities were diabetes mellitus (33.6%) and hypertension (13.3%). The most common acute decompensation was gastrointestinal bleeding (70.9%), followed by hepatic encephalopathy (26.9%) and ascites (23.6%). Pneumonia was the most common infection (22%). According to the European Association for the Study of the Liver (EASL) definition for Acute-on-Chronic Liver Failure (ACLF) [17], approximately 75% of our patients had no ACLF at hospital admission.

**Table 1:** Baseline characteristics of the study population

Characteristic	Entire cohort (n = 241)	Discharged alive (n = 212)	Died during hospitalization (n = 29)
Age (year), mean ± SD	53.4 ± 12.03	52.25 ± 11.5	61.5 ± 13.02
Sex, n (%)			
Male	179 (74.3)	162 (76.4)	17 (58.6)
Female	62 (25.7)	50 (23.6)	12 (41.4)
Etiology, n (%)			
Hepatitis B	93 (38.6)	84 (39.6)	9 (31)
Hepatitis C	70 (29)	57 (26.9)	13 (44.8)
Hepatitis B & C	5 (2.1)	5 (2.4)	0 (0)
NAFLD	3 (1.3)	3 (1.4)	0 (0)
Alcohol	1 (0.04)	1 (0.5)	0 (0)
Cryptogenic	69 (28.6)	62 (29.25)	7 (24.14)
Child–Pugh, n (%)			
A	53 (22)	52 (24.5)	1 (3.4)
B	96 (40)	91 (42.9)	5 (17.2)
C	92 (38)	69 (32.5)	23 (79.3)
Score, median (min–max)	9 (5–15)	8 (5–15)	11 (6–14)
HCC (within the Milan Criteria), n (%)	4 (1.7)	4 (1.89)	0 (0)
Comorbidities, n (%)			
Diabetes mellitus	81 (33.6)	67 (31.6)	14 (48.3)
Hypertension	32 (13.3)	30 (14.1)	2 (6.9)
Charlson comorbidities, n (%)			
None	138 (57.3)	124 (58.5)	14 (48.3)
1	77 (31.9)	66 (31.1)	11 (37.9)
2	22 (9.1)	18 (8.5)	4 (13.8)
≥ 3	4 (1.6)	4 (1.9)	0 (0)
Gastrointestinal bleeding, n (%)	171 (70.9)	157 (74)	14 (48.3)
Hepatic encephalopathy, n (%)			
Grade I–II	49 (20.3)	36 (16.9)	13 (44.8)
Grade III–IV	16 (6.6)	14 (6.6)	2 (6.9)
Ascites, n (%)	57 (23.6)	46 (21.7)	11 (37.9)
Bacterial infection, n (%)	61 (25.3)	41 (19.3)	20 (68.9)
Pneumonia	53 (22)	34 (16)	19 (65.5)
Spontaneous bacterial peritonitis	8 (3.3)	5 (2.3)	3 (10.3)
Urinary tract infection	4 (1.6)	3 (1.4)	1 (3.4)
Empyema	1 (0.4)	1 (0.47)	0 (0)
Skin infection	4 (1.7)	4 (1.88)	0 (0)
Previous acute decompensation, n (%)	48 (20)	44 (20.7)	4 (13.8)
MAP (mmHg), mean ± SD	83.7 ± 14.7	84.2 ± 14.6	80.7 ± 14.9
SpO_2_/FiO_2_ ratio	461.9 (84.21–476.2)	466.6 (84.21–476.2)	306.2 (104.21–476.2)
Leucocyte count (× 10^3^/µL)	8.4 (1.11–42.1)	8.06 (1.11–42.1)	13.2 (3.2–30.7)
Neutrophil count (× 10^3^/µL)	5.9 (0.62–37.05)	5.8 (0.62–37.05)	10.2 (1.71–27.27)
Albumin (mg/dl), mean ± SD	2.7 ± 0.6	2.8 ± 0.6	2.5 ± 0.5
Total bilirubin (mg/dl)	2.02 (0.4–57.8)	1.9 (0.4–40.9)	4.9 (0.8–57.8)
Creatinine (mg/dl)	0.9 (0.13–9.3)	0.9 (0.3–5.7)	1.3 (0.13–9.3)
Sodium (mg/dl), mean ± SD	134.9 ± 6.4	135.5 ± 6.2	130.8 ± 6.4
Prothrombin time (sec)	13.3 (9.9–120)	12.8 (9.9–120)	15.4 (11–42.6)
MELD	12.8 (6.4–43.5)	12.4 (6.4–39.6)	22.8 (8.4–43.5)
MELD-Na	15.7 (6.4–43.3)	14.7 (6.4–39.6)	25.9 (12.6–43.3)
CLIF-C OF score	7 (6–13)	7 (6–13)	9 (6–13)
ACLF grade, n (%)^a^			
No ACLF	180 (74.7)	169 (79.7)	11 (37.9)
Grade 1	44 (18.3)	33 (15.6)	11(37.9)
Grade 2	12 (5)	6 (2.8)	6(20.7)
Grade 3	5 (2.1)	4 (1.9)	1 (3.4)
Causes of death, n (%)			
Septic shock			13 (44.8)
Hypovolemic shock			2 (6.9)
Respiratory failure			8 (27.6)
Liver failure			1 (3.4)
Cardiac arrest			3 (10.3)
Cause unknown			2 (6.9)

Data are presented as medians (minimum–maximum) unless otherwise stated. MAP, Mean arterial pressure; MELD-Na, Model for end-stage liver disease-sodium; CLIF-C OF, Chronic liver failure-consortium organ failure; ACLF, Acute-on-chronic liver failure; NAFLD, Nonalcoholic fatty liver disease; HCC, Hepatocellular carcinoma; SD, Standard deviation

^a^ACLF grade and classification based on EASL clinical practice guideline [17]

### The independent predictors of in-hospital mortality

For logistic regression analysis, variables with skewed distribution were transformed into natural logarithm (neutrophil count, total bilirubin, creatinine) or 1/square form (prothrombin time). The SpO2/FiO2 ratios were converted into categorical data using the median value as the cut-off (< 460 or ≥ 460). The bivariate analysis showed that age, hepatic encephalopathy, bacterial infection, the SpO_2_/FiO_2_ ratio, the neutrophil count, the sodium level, albumin, the total bilirubin level, the creatinine level, and prothrombin time were significant predictors of in-hospital mortality. The multivariate analysis showed that age (adjusted OR: 1.09; 95% CI: 1.03–1.14), bacterial infection (adjusted OR: 6.25; 95% CI: 2.31–16.92), the total bilirubin level (adjusted OR: 3.01; 95% CI: 1.85–4.89) and the creatinine level (adjusted OR: 2.70; 95% CI: 1.20–6.05) were independent predictors of in-hospital mortality in acutely decompensated liver cirrhosis patients. The results are summarized in Table 2.

**Table 2:** Bivariate and multivariate analyses of in-hospital mortality

Variables	Bivariate analysis		Multivariate analysis	
	OR (95% CI)	p-value	Adjusted OR (95% CI)	p-value
Age (years)	1.07 (1.03–1.11)	< 0.001	1.09 (1.03–1.14)	0.001
Charlson Comorbidities	1.23 (0.74–2.03)	0.416		
Previous acute decompensation	0.61 (0.20–1.85)	0.383		
Hepatic encephalopathy	3.47 (1.56–7.68)	0.002		
Bacterial infection	9.27 (3.93–21.84)	< 0.001	6.25 (2.31–16.92)	< 0.001
MAP	0.98 (0.96–1.01)	0.234		
SpO_2_/FiO_2_ ratio	4.60 (1.88–11.25)	0.001		
Neutrophil count (ln)	2.89 (1.54–5.43)	0.001		
Sodium	0.90 (0.85–0.96)	< 0.001		
Albumin	0.42 (0.21–0.80)	0.009		
Total bilirubin (ln)	2.58 (1.77–3.77)	< 0.001	3.01 (1.85–4.89)	< 0.001
Creatinine (ln)	3.59 (1.79–7.18)	< 0.001	2.70 (1.20–6.05)	0.016
Prothrombin time (l/sqr)	2.17 (1.05–4.46)	< 0.001		

ln, natural logarithm; MAP, mean arterial pressure

### Development of scoring systems

#### Logistic scoring system

Based on the multivariate logistic regression analysis (Table 3), we established the following predictive model:

**Table 3:** Multivariate logistic regression analysis with backward stepwise method (n = 241)

Variables	B	SE	Z	p-value
Age	0.086	0.025	3.42	0.001
Bacterial infection	1.834	0.507	3.61	< 0.001
Total bilirubin (ln)	1.101	0.248	4.44	< 0.001
Creatinine (ln)	0.994	0.411	2.42	0.016
Constant	− 9.299	1.762	− 5.28	< 0.001

ln: natural logarithm, SE: standard error, Z: B/SE

$${\text{Logistic}}\,{\text{score}}\, = \,0.086\, \times \,{\text{age }}\left( {{\text{year}}} \right)\, + \,1.834\, \times \,{\text{bacteria}}\,{\text{lnfection}}\,\left( {0\,{\text{if}}\,{\text{absent}},{\text{ }}1\,{\text{if}}\,{\text{present}}} \right)\, + \,1.101\, \times \,\ln {\text{ }}\left( {{\text{total}}\,{\text{bilirubin}},\,{\text{mg/dL}}} \right)\, + \,0.994\, \times \,\ln {\text{ }}\left( {{\text{creatinine}},\,{\text{mg/dL}}} \right){-}9.299$$

**Fig. 1:**
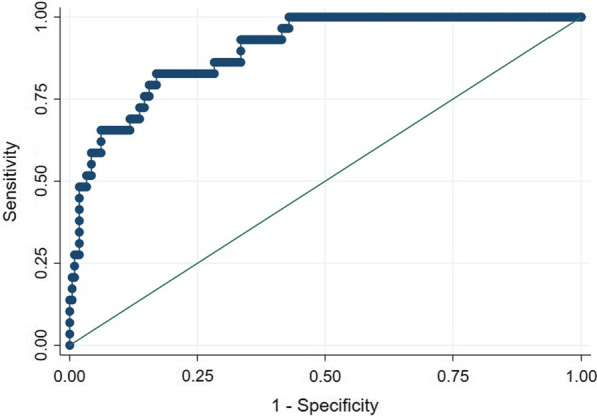
Receiver operating characteristic curve for the logistic score. AUROC: 0.899

The range for the total logistic score was -9.520–5.670. The probability of in-hospital mortality was calculated by equation: p = 1/(1 + exp(-y)), with y = 0.999 × logistic score—0.019. This logistic score produced an AUROC of 0.899 (95% CI: 0.846–0.952), and a cut-off value ≥ -1.8184 had a sensitivity of 82.8% and specificity of 83% (Fig. 1). The observed and expected probabilities of in-hospital mortality were similar (Additional file 1: Table S1, Figure S2). The Hosmer–Lemeshow test indicated a good fit (x^2^ = 12.60; *p* = 0.1265). The AUROC of the logistic score was significantly better than those of MELD, MELD-Na or CLIF-C OF score (0.826 [*p* = 0.0432], 0.831 [*p* = 0.0142] and 0.752 [*p* = 0.0005] respectively) (Table 6).

#### Additive scoring system

**Fig. 2:**
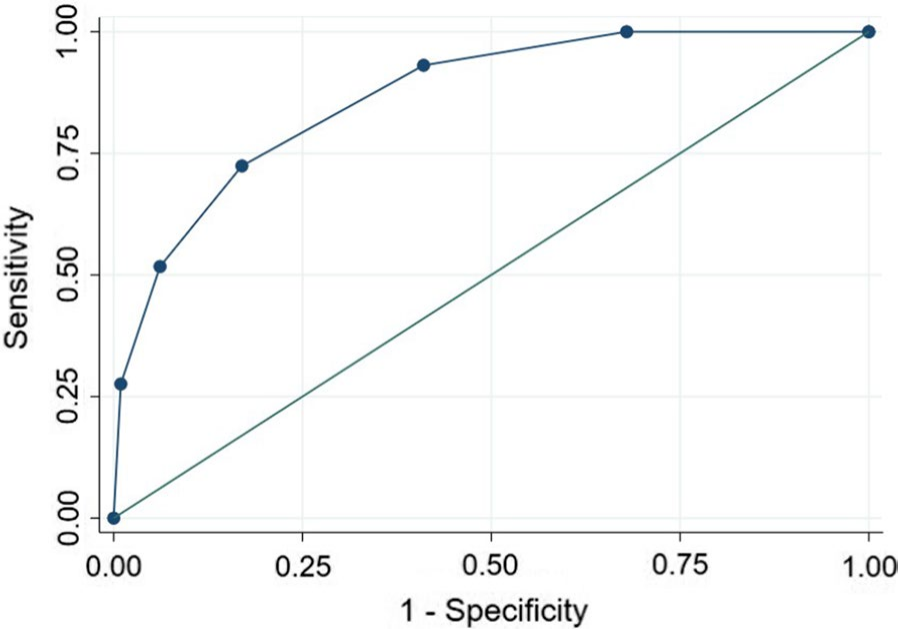
Receiver operating characteristic curve for the additive score. AUROC: 0.868

For the development of the additive scoring system, continuous variables were converted into categorical variables: age < 57 or ≥ 57 years, total bilirubin < 3 or ≥ 3 mg/dl, and creatinine < 1.1 or ≥ 1.1 mg/dl. Based on the multivariate logistic regression analysis (Table 4), the scores for each variable were determined by dividing the z value (B/SE) by the smallest value (2.27) and rounding to the nearest 0.5. The additive scoring system for in-hospital mortality is presented in Table 5. The total additive score ranges from 0 (no risk factors are present) to 5 (the subject has all risk factors). The probability of in-hospital mortality was calculated by equation: p = 1/(1 + exp(− y)), with y = 1.132 × additive score – 4.670. The discriminatory power of the additive score was good (AUROC: 0.868; 95% CI: 0.806–0.930), and a cut-off ≥ 3 had a sensitivity of 72.4% and a specificity of 83% (Fig. 2). The observed and expected probabilities of in-hospital mortality across different levels of the additive score were similar (Additional file 1: Table S2, Figure S3). The Hosmer–Lemeshow test also indicated a good fit (x^2^ = 1.17; *p* = 0.7595). The AUROC of the additive score was significantly better than that of CLIF-C OF score (*p* = 0.0151), but not significantly better than those of MELD (*p* = 0.3097) and MELD-Na (*p* = 0.2451) (Table 6).

**Table 4:** Multivariate logistic regression analysis of the additive score

Variables	B	SE	z	p-value
Age	1.116	0.491	2.27	0.023
Bacterial Infection	1.935	0.495	3.91	< 0.001
Total bilirubin	1.400	0.507	2.76	0.006
Creatinine	1.509	0.497	3.03	0.002
Constant	− 4.859	0.650	− 7.48	< 0.001

SE: standard error; Z: B/SE

**Table 5:** Additive scoring system for in-hospital mortality

Variables	Category	Score
Age	< 57 years old	0
	≥ 57 years old	1
Bacterial infection	No	0
	Yes	2
Total bilirubin (mg/dL)	< 3	0
	≥ 3	1
Creatinine (mg/dL)	< 1.1	0
	≥ 1.1	1

**Table 6:** Discrimination ability of logistic and additive score as compared to MELD, MELD-Na, CLIF-C OF (n = 241)

Score	AUROC	95%CI	*p-*value	
			*vs.* Logistic score	*vs.* Additive score
Logistic	0.899	0.846–0.952		
Additive	0.868	0.806–0.930		
MELD	0.826	0.751–0.902	0.0432	0.3097
MELD-Na	0.831	0.758–0.903	0.0142	0.2451
CLIF-C OF	0.752	0.656–0.847	0.0005	0.0151

MELD, Model for end-stage liver disease; MELD-Na, Model for end-stage liver disease-sodium; CLIF-C OF, Chronic liver failure-consortium organ failure

## Discussion

Previously, we reported the long-term prognosis of hospitalized cirrhotic patients [18]. This study involved 241 adult patients with acutely decompensated liver cirrhosis and an in-hospital mortality rate of 12.03%. We identified independent predictors of in-hospital mortality and then developed predictive scoring systems for daily clinical application.

The majority of the patients in this study were male, with a mean age of 53 years old and Child–Pugh class B or C cirrhosis. This is similar to the characteristics of hospitalized liver cirrhosis patients reported by multinational prospective studies in Europe (the CANONIC-EASL-CLIF/Acute-on-Chronic Liver Failure in Cirrhosis study) and North America (NACSLED/North American Consortium for the Study of End-Stage Liver Disease study) [7, 19, 20]. In the CANONIC and NACSLED studies, the most common aetiologies of liver cirrhosis were alcohol consumption and hepatitis C. However, this study found that the most common etiology was hepatitis B. This is consistent with the epidemiological data of hepatitis B infection in Indonesia. One-third of the subjects also had diabetes mellitus, similar to that reported in the NACSLED study.

In this study, 70.95% of the subjects presented with gastrointestinal bleeding, whereas in the CANONIC study, most of the liver cirrhosis patients were treated for hepatic encephalopathy and ascites [19]. The high proportion of gastrointestinal bleeding in this study is thought to be associated with the degree of increased portal venous pressure, given that portal vein pressure measured indirectly by the hepatic venous pressure gradient (HVPG) is significantly higher in Child–Pugh B and C cirrhosis than in Child–Pugh A cirrhosis [21]. A total of 25.31% of subjects had bacterial infections, with the most common types being pneumonia, spontaneous bacterial peritonitis, and urinary tract infection, similar to that previously reported [22].

The in-hospital mortality rate in this cohort was 12.03%, which was relatively higher than that previously reported [6, 23–26]. A systematic analysis of the mortality rates of liver cirrhosis patients in 187 countries by Mokdad et al. indicated a 22% decline in the 1980–2010 period in developed countries in Europe, China, and the United States, while in Indonesia, this figure had increased. Lower mortality rates in developed countries were associated with improved preventive measures, such as hepatitis screening for blood donors, hepatitis B vaccination, and alcohol consumption restrictions [3].

This study found that age, bacterial infection, bilirubin, and creatinine level were all predictors of in-hospital mortality in acutely decompensated liver cirrhosis patients. Similar results were also reported in other studies [6, 7, 20, 24]. These studies have consistently shown a significant association between age and mortality due to liver cirrhosis. However, in line with previous studies, we found that age was not the major predictor of in-hospital mortality in acutely decompensated liver cirrhosis [8, 20, 24].

Bacterial infection is commonly diagnosed in 20–25% of liver cirrhosis patients [27]. Similar to that in previous studies, we found that bacterial infection had a strong association with in-hospital mortality (adjusted OR: 6.25, 95% CI: 2.31–16.92, p < 0.001) [19, 27–29]. Therefore, early diagnosis and treatment of bacterial infection should be routinely performed in patients with acutely decompensated liver cirrhosis. The diagnosis of bacterial infection using leukocyte count is often difficult in liver cirrhosis patients due to hypersplenism. In addition, leukocytosis occurs in only < 50% of patients with infection. A study by Li Y et al. showed that the neutrophil percentage was associated with 90-day mortality in advanced liver cirrhosis patients with bacterial infection [16]. In our study, the median neutrophil count was higher in the nonsurvivors than in the survivors, and it was significantly associated with in-hospital mortality in the univariate analysis but not in the multivariate analysis. Other studies have shown that mortality due to liver cirrhosis is associated with decreased chemotaxis and the phagocytosis capacity of neutrophils [30].

The increased systemic inflammatory response in acutely decompensated liver cirrhosis patients could cause liver and extrahepatic organ dysfunction. The bilirubin level is routinely measured as a marker of liver cirrhosis. The Asian Pacific Association for the Study of the Liver (APASL) consensus on acute-on-chronic liver failure (ACLF) suggested that a bilirubin level of 5–10 mg/dL was associated with a mortality rate of 38% [31]. The CANONIC study showed that a bilirubin level ≥ 12 mg/dL was associated with a 28-day mortality rate of 15% [19]. A study by López-Velázquez et al. showed that a bilirubin level ≥ 3.45 mg/dL at admission predicted one-week mortality in decompensated cirrhotic patients with ACLF [32]. Accordingly, we also found that the total bilirubin level was an independent predictor of in-hospital mortality.

Renal dysfunction in acutely decompensated liver cirrhosis patients may occur as a result of hypovolemia or bleeding, bacterial infection, hepatorenal syndrome, renal parenchymal abnormalities, use of drugs or contrast agents, or a combination of these factors. The in-hospital mortality rate in acutely decompensated liver cirrhosis patients with type 1 hepatorenal syndrome was higher than those with bleeding or hypovolemia [33]. Our study showed that each incremental increase in creatinine increased the risk of in-hospital mortality by up to three times, independent of bacterial infection. This indicates that antibiotic therapy alone may not be sufficient to reduce the risk of in-hospital mortality. Therefore, close monitoring of renal function is imperative in acutely decompensated liver cirrhosis patients. Currently, international consensus recommends albumin for all cirrhotic patients with spontaneous bacterial peritonitis and hepatorenal syndrome to reduce mortality and improve renal function [17].

The economic burden of treating decompensated liver cirrhosis patients is very high, and prognostic models or scoring systems could help clinicians deliver more cost-effective care to these patients [34]. In this study, we developed predictive scoring systems using clinical data obtained routinely at the time of hospital admission in both additive and logistic forms. Both the logistic and additive scoring systems showed good discriminatory powers. The simple additive form is calculated by adding up scoring points, so it can be performed bedside in a resource-limited clinical setting. The logistic form needs complex calculations with slightly improved accuracy. Therefore, the logistic score can be integrated into hospital information technology system for more precise, predictive analysis. Furthermore, we found that the logistic score showed better discrimination ability compared to other scores such as MELD, MELD-Na and CLIF-C OF.

There are several limitations to our study. This study was a single-centre, retrospective cohort study and is therefore susceptible to information bias. In daily clinical practice, using our scoring systems at the time of hospital admission could help clinicians with an early screening of high-risk patients. However, further studies are needed to see whether a serial assessment of the scoring systems throughout hospitalization also correlate with mortality outcome. Previous studies showed that in high-risk patients with variceal bleeding (HVPG ≥ 20 mmHg, Child–Pugh B with active bleeding or Child–Pugh C ≤ 13), pre-emptive TIPSS improved the long-term mortality rate [35, 36]. Pre-emptive TIPSS was also associated with decreased in-hospital mortality [37]. Due to resources limitation at our institution, none of our patients were treated with TIPSS. Further studies are needed to confirm whether our scoring systems could also select those high-risk patients who are candidates for pre-emptive TIPSS. Further validation of our proposed predictive scoring systems in different populations is needed to confirm our findings.

## Conclusion

In conclusion, the in-hospital mortality rate of acutely decompensated liver cirrhosis patients in Indonesia is still high. Age, bacterial infection, total bilirubin, and creatinine levels were independent predictors of in-hospital mortality. The scoring systems for daily clinical use, in the form of a logistic score and an additive score, can be used to predict in-hospital mortality in acutely decompensated liver cirrhosis patients.

## Supplementary Information

**Additional file 1. Figure S1**. The flow chart of the cohort study.** Table S1**. Contingency tables for the Hosmer-Lemeshow test for the logistic score.** Table S2**.  Contingency tables for the Hosmer-Lemeshow test for the additive score. **Figure S2**. Calibration plot for the logistic score (Spearman’s rho = 0.9147; p = 0.0002.** Figure S3**. Calibration plot for the additive score (Spearman’s rho = 0.9747; p = 0.0048).

## Data Availability

The datasets analysed during the current study are available from the corresponding author on reasonable request.

## Abbreviations

ACLF: Acute-on-chronic liver failure
APASL: Asian Pacific Association for the Study of the Liver
AUROC: Area under the ROC curve
CANONIC: Chronic liver failure (CLIF) acute-on-chronic liver failure in cirrhosis
CLIF-AD: Chronic liver failure-acute decompensation
CLIF-C OF: Chronic liver failure-consortium organ failure
EASL-CLIF: European Association for the Study of the Liver-Chronic Liver Failure
CT: Computed tomography
HCC: Hepatocellular carcinoma
HIV: Human immunodeficiency virus
HVPG: Hepatic venous pressure gradient
MAP: Mean arterial pressure
MELD: Model for End-Stage Liver Disease
MELD-Na: MELD-sodium
MRI: Magnetic resonance imaging
NACSLED: North American Consortium for the Study of End-Stage Liver Disease
NAFLD: Nonalcoholic fatty liver disease
OR: Odds ratio
ROC: Receiver operating characteristic
TIPSS: Transjugular intrahepatic portosystemic shunt
