# Supplementary material for: Predictive scoring systems for in-hospital mortality due to acutely decompensated liver cirrhosis in Indonesia
Source: BMC Gastroenterol. 2021 Oct 20;21:392. doi: 10.1186/s12876-021-01972-6 (PMC8529806; doi:10.1186/s12876-021-01972-6)
Supplement: Supplementary file 1 — Additional file 1. Figure S1. The flow chart of the cohort study. Table S1. Contingency tables for the Hosmer-Lemeshow test for the logistic score. Table S2. Contingency tables for the Hosmer-Lemeshow test for the additive score. Figure S2. Calibration plot for the logistic score (Spearman’s rho = 0.9147; p = 0.0002. Figure S3. Calibration plot for the additive score (Spearman’s rho = 0.9747; p = 0.0048). [file 12876_2021_1972_MOESM1_ESM.docx]

**Predictive Scoring Systems for In-hospital Mortality due to Acutely Decompensated Liver Cirrhosis in Indonesia**

Saut Horas H. Nababan^1,*^, Arif Mansjoer^2^, Achmad Fauzi^3^, Rino A Gani^1^

^1^ Hepatobiliary Division, Internal Medicine Department, Cipto Mangunkusumo National General Hospital, Faculty of Medicine, Universitas Indonesia, Jl. Diponegoro No. 71 Jakarta Indonesia 10430.

^2^ Clinical Epidemiology Unit, Internal Medicine Department, Cipto Mangunkusumo National General Hospital, Faculty of Medicine, Universitas Indonesia, Jl. Diponegoro No.71 Jakarta Indonesia 10430.

^3^ Gastroenterology Division, Internal Medicine Department, Cipto Mangunkusumo National General Hospital, Faculty of Medicine, Universitas Indonesia, Jl. Diponegoro No.71 Jakarta Indonesia 10430.

*Corresponding author:

Saut Horas H Nababan. Email: sautnbbn83@gmail.com.

Hepatobiliary Division, Internal Medicine Department, Cipto Mangunkusumo National General Hospital, Faculty of Medicine, Universitas Indonesia, Jl. Diponegoro No.71 Jakarta Indonesia 10430. Tel.: +62 21-31900924, Fax: +62 21-3918842

**Supplementary Tables and Figures**

Acutely decompensated liver cirrhosis (January 2016 - December 2019)

(N=894)

Exclusion (n=351)

- HIV coinfection (n= 31)
- Immunosuppressive treatment (n=5)
- Advanced HCC (beyond Milan criteria) (n=255)
- < 24 hours hospitalization (n= 60)

241 patients included in the analysis

Lost or incomplete data (n=302)

**Figure S1.** The flow chart of the cohort study. HIV, human immunodeficiency virus; HCC, hepatocellular carcinoma**.**

**Table S1. Contingency tables for the Hosmer-Lemeshow test for the logistic score**

|  |  | **Death = 1** |  | **Death = 0** |  |  |
| --- | --- | --- | --- | --- | --- | --- |
| **Group** | **Probability** | **Observed** | **Expected** | **Observed** | **Expected** | **Total** |
| 1 | 0.0026 | 0 | 0 | 25 | 25 | 25 |
| 2 | 0.0057 | 0 | 0.1 | 24 | 23.9 | 24 |
| 3 | 0.0112 | 0 | 0.2 | 24 | 23.8 | 24 |
| 4 | 0.0192 | 0 | 0.4 | 24 | 23.6 | 24 |
| 5 | 0.0299 | 0 | 0.6 | 24 | 23.4 | 24 |
| 6 | 0.0528 | 4 | 0.9 | 20 | 23.1 | 24 |
| 7 | 0.0908 | 1 | 1.7 | 23 | 22.3 | 24 |
| 8 | 0.1929 | 4 | 3.3 | 20 | 20.7 | 24 |
| 9 | 0.4016 | 5 | 6.6 | 19 | 17.4 | 24 |
| 10 | 0.9905 | 15 | 15.1 | 9 | 8.9 | 24 |

.

**Table S2. Contingency tables for the Hosmer-Lemeshow test for the additive score**

|  |  | **Death = 1** |  | **Death = 0** |  |  |
| --- | --- | --- | --- | --- | --- | --- |
| **Group** | **Probability** | **Observed** | **Expected** | **Observed** | **Expected** | **Total** |
| 1. | 0.0093 | 0 | 0.6 | 68 | 67.4 | 68 |
| 2. | 0.0282 | 2 | 1.7 | 57 | 57.3 | 59 |
| 3. | 0.0827 | 6 | 4.7 | 51 | 52.3 | 57 |
| 4. | 0.2185 | 6 | 6.3 | 23 | 22.7 | 29 |
| 5. | 0.7290 | 15 | 15.7 | 13 | 12.3 | 28 |


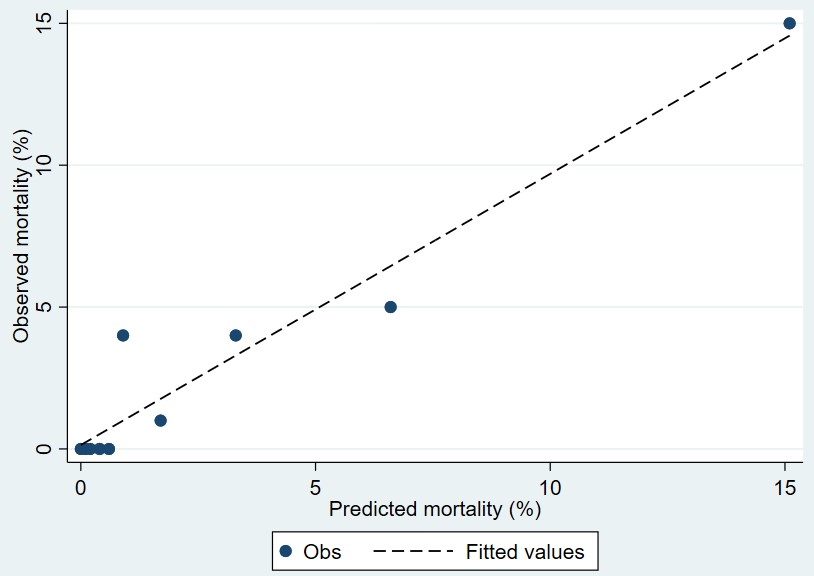


**Figure S2. Calibration plot for the logistic score (Spearman's rho = 0.9147; p = 0.0002)**


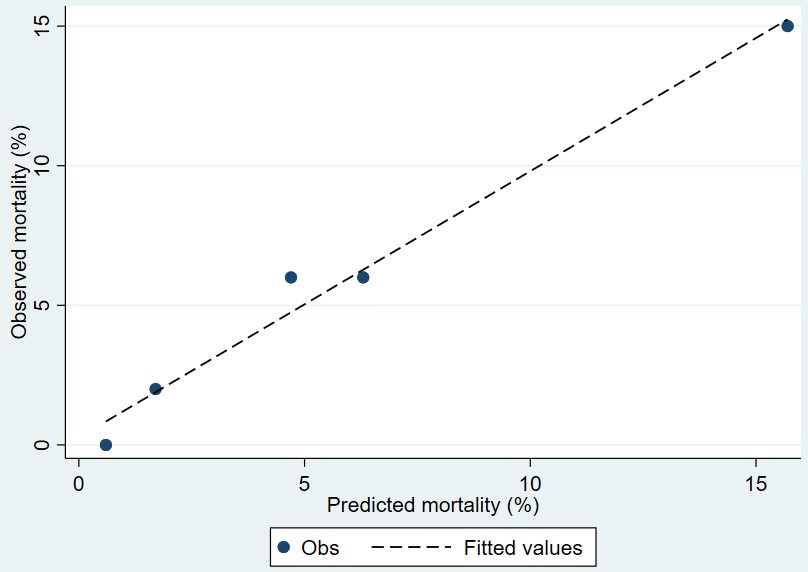


**Figure S3. Calibration plot for the additive score (Spearman's rho = 0.9747; p = 0.0048).**
